# The Incidence and Risk Factors of Hip Fracture after Liver Transplantation (LT): A Nationwide Population-Based Study

**DOI:** 10.1155/2019/5845709

**Published:** 2019-12-24

**Authors:** Yung-Cheng Chiu, Pei-Shao Liao, Yi-Ting Chou, Cheng-Li Lin, Chih-Hung Hung, Che-Chen Lin, Chieh-Cheng Hsu, Horng-Chaung Hsu, Jyun-Ming Huang, Yang-Yi Wang, Shu-Jui Kuo

**Affiliations:** ^1^School of Medicine, China Medical University, Taichung, Taiwan; ^2^Department of Orthopedic Surgery, China Medical University Hospital, Taichung, Taiwan; ^3^Department of Education, China Medical University Hospital, Taichung, Taiwan; ^4^Management Office for Health Data, China Medical University Hospital, Taichung, Taiwan; ^5^Department of Orthopedic Surgery, Kaohsiung Chang Gung Memorial Hospital and Chang Gung University College of Medicine, Kaohsiung, Taiwan; ^6^Department or Surgery, China Medical University Hospital, Taichung, Taiwan; ^7^Department of Education, National Taiwan University Hospital, Taipei, Taiwan

## Abstract

**Background:**

Osteoporosis and fragility fracture are the major complications after liver transplantation (LT). The aim of the study was to determine the incidence and risk factors of hip fracture after LT.

**Methods:**

We conducted a retrospective population-based cohort study, enrolling the patients receiving LT between January 1999 and December 2010. Control subjects were randomly matched to every recipient by age and sex by 1 : 10 ratios.

**Results:**

During the follow-up period, 17 recipients (0.77%) and 70 (0.32%) control subjects suffered from hip fractures. The incident rates (per 10000 person-years) were 21.49 for recipients and 7.52 for controls (adjusted hazard ratio = 2.71; 95% confidence interval = 1.21–6.05). The cumulative incidence of hip fracture was significantly higher among the recipients (*p* < 0.0001). Among the recipients, the subjects aged >65 years at transplantation and with pretransplant steroid use are more susceptible to posttransplant hip fracture. Immunosuppressive agents did not significantly affect the risk of hip fracture among recipients.

**Conclusions:**

Liver transplantation is a risk factor for hip fractures. Aged >65 years at transplantation and pretransplant steroid use are risk factors for posttransplant hip fractures among the recipients.

## 1. Introduction

Liver transplantation (LT) is the most effective treatment for patients with decompensated chronic liver disease and significantly improves both quality of life and survival of the recipients [1]. However, osteoporosis and fragility fracture substantially threatened the quality of life and the survival of the recipients [2–4]. The fractures occur mainly during the first 6 to 12 months following LT, with the ribs and vertebrae being the most common sites [5]. Despite the fact that hip fractures have been notoriously associated with considerable disability, costs, and risk of mortality, the correlation between LT and hip fracture was undetermined at present [6]. Previous studies have been limited by the small sample size and the short follow-up period [5, 7, 8].

Because of the devastating outcome after the hip fracture, we aimed to determine the pertinent epidemiologic information, including incidence and risk factors, about the hip fracture after LT.

## 2. Materials and Methods

### 2.1. Database

This study was approved by the Ethics Review Board of China Medical University (CMU-REC-101-012).

The Taiwanese National Health Insurance (NHI) program offers compulsory comprehensive health insurance in Taiwan since 1995. All contracted medical institutions submit computerized claim documents for medical expenses. Data analyzed in our study were obtained from the National Health Insurance Research Database (NHIRD) (available at http://www.doh.gov.tw/EN2006/index_EN.aspx (in English)). The NHIRD covers all claims of Taiwan NHI, and it is one of the largest and most comprehensive databases in the world. The database included the information about the registry for beneficiaries, the record of historical diseases, and the registry for drug prescriptions and other medical services. The Taiwanese government removed the original identification number to safeguard the privacy for the insured citizens and provided a scrambled and anonymous identification number to link the data for each insured citizen before releasing for research.

The ICD-9-CM (International Classification of Diseases, 9th Revision, Clinical Modification) system was utilized as the disease coding system in NHIRD. The history of liver transplantation and end-stage renal disease (ESRD) was obtained from the files of registration for catastrophic illness. The history of fracture and other comorbidities was collected from outpatient and inpatient files.

### 2.2. Patient Selection and Definition

We conducted a retrospective population-based cohort study and collected patients receiving liver transplantations (ICD-9-CM V42.7 and 996.82) between January 1999 and December 2010 in the NHIRD database. The index date for the recipient cohort was the date of transplantation surgery. The control subjects without any transplantation history were randomly matched to every recipient by age (per 5 years) and sex at the 1 : 10 ratio. The index date for the control cohort was defined as the date of transplantation surgery of the matched recipient. The subjects with cancer history (ICD-9-CM 140-208), accident indicative of high energy trauma (presence of E coding), and previous hip fracture history (ICD-9-CM 820) were all excluded [9]. We also excluded the subjects with the history of antiresorptive and/or anabolic therapy at index date. All of the recruited subjects were followed from the index date to the onset of hip fracture, withdrawal from insurance, or December 31, 2011.

Baseline comorbidities including coronary artery disease (CAD), diabetes mellitus (DM), epilepsy, hypertension, osteoporosis, stroke, end-stage renal disease (ESRD), hepatitis B virus infection (HBV), hepatitis C virus infection (HCV), and oral or intravenous steroid use > 30 days before recruitment were included for analysis. The diagnosis of osteoporosis would only be coded by the Taiwanese physician if the patient had the *T* score value of less than −2.5 in the bone densitometry assay [9]. We also analyzed the recipients to determine the risk factors for hip fracture after transplantation. Immunosuppressive agents, including tacrolimus, everolimus, mycophenolate mofetil (MMF), and cyclosporin, were analyzed for its impact on the fracture occurrence among the recipients.

### 2.3. Statistical Analyses

The continuous variables were expressed as mean ± standard deviation, whilst the categorical variables were expressed as number and percentage. We assessed the significance of between-group differences via Student's *t*-test for continuous variables and chi-square test for categorical variables. We calculated the incidence of hip fractures (case per 10000 person-years) by dividing the total number of hip fractures by the sum of follow-up years. The Kaplan–Meier method was utilized to demonstrate the cumulative incidence for the two groups with the significance of difference assessed by the log rank test. We construct the single-variant and multivariant Cox proportional hazard models to calculate the hazard ratios (HRs) and 95% confidence intervals (CIs) of the parameters of transplantation, demographic factors, and comorbidities in order to evaluate the impact of these factors on the fracture occurrences.

All of the statistical analyses were performed by SAS 9.4 software (SAS Institute, Cary, NC, USA). The cumulative incidence curves were drawn by *R* software (*R* Foundation for Statistical Computing, Vienna, Austria). All of the analyses were performed two-sided, and *p* < 0.05 was considered statistically significant.

## 3. Results

We enrolled 2201 liver recipients, and each recipient was matched by 10 sex-and-age matched controls. The composition of age and sex was homogenous between the two groups. Besides osteoporosis, recipients were prone to suffer from more baseline comorbidities, including DM, epilepsy, hypertension, osteoporosis, stroke, ESRD, HBV infection, HCV infection, and prerecruitment steroid use (Table 1).

**Figure 1:**
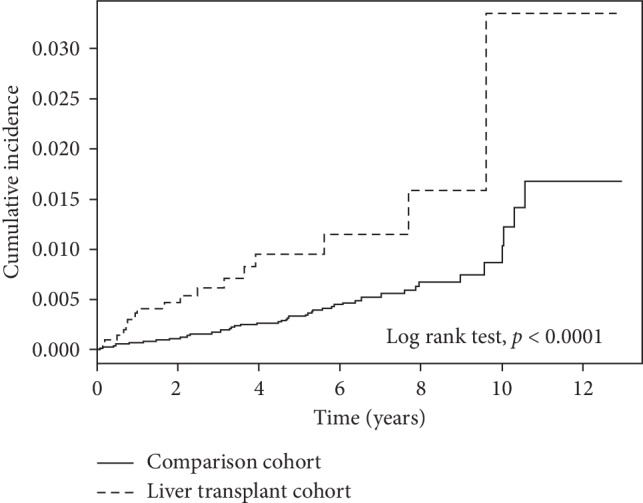
Cumulative incidence of hip fracture among liver recipients and control subjects. The cumulative incidence in the liver transplant cohort was significantly higher than that in the comparison cohort (*p* < 0.0001 by log rank test).

During the follow-up period, 17 recipients (17/2201 = 0.77%) and 70 controls (70/22010 = 0.32%) suffered from hip fractures. The incident rates were 21.49 for the recipients and 7.52 for the controls (per 10000 person-years, adjusted HR = 2.71, 95% CI = 1.21–6.05). Under the Kaplan–Meier analysis, the cumulative incidence of hip fracture was significantly higher among recipients than among controls (*p* < 0.0001 by log rank test) (Figure 1). The interval between transplantation and hip fracture was 2.6 ± 2.7 years, and the interval between recruitment and hip fracture was 3.7 ± 2.8 years among control subjects (*p*=0.18).

All of the 2201 liver recipients and 22010 controls were included in our hazard model for single-variant and multivariant analysis. Transplantation, age, stroke, and HCV infection before recruitment correlated with higher risk for hip fracture under both single-variant and multivariant analysis (Table 2).

We tried to determine the risk factors for posttransplant hip fracture among the recipients. Among the recipients, the subjects aged more than 65 years old at transplantation are 14.64 times (adjusted HR = 14.64, 95% CI = 1.47∼145) more likely to suffer from hip fracture than the recipients undergoing transplantation at the age of <45 years. It is noteworthy that all of the recipients suffering from posttransplant hip fracture had the history of steroid use >30 days before the transplantation. In other words, none of the recipients without pretransplant steroid use developed posttransplant hip fracture in our series. There was no significant impact of immunosuppressive agents after transplantation, including tacrolimus, everolimus, mycophenolate mofetil (MMF), and cyclosporin, on the occurrence of hip fracture (Table 3).

## 4. Discussion

Osteoporosis is a grave complication after liver transplantation [2, 4]. While hip fracture is considered as the osteoporosis-related fragility fracture, the correlation between LT and hip fracture is not validated at present. The incidence rates of hip fracture after LT are variable among different series [5, 7, 8].

There were series reporting the occurrence of fracture events after Leidig-Bruckner et al. recruited 130 recipients and followed for 7 years. Nine recipients suffered from nonvertebral fractures [7]. Guichelaar et al. followed 360 recipients for 8 years, and the cumulative incidence of fracture other than spine, rib, and pelvis was 4.2% at 1 year and 9.5% at 8 years [8]. Both studies did not specify the occurrence of hip fractures. Premaor et al. followed 531 recipients for 10 years and recognized 1 hip fracture only [5]. All of the above studies did not recruit controls for comparison and did not exhibit higher incidence of hip fracture among the recipients. In our study, we established the Cox regression model pooling 2201 recipients and 22010 age-and-sex matched controls together. Under our model, we identified that LT, age (>65 years old), stroke, and HCV infection were associated with higher risk for hip fracture. Some of the factors have been identified by previous studies. Previous epidemiologic study has shown that hip fractures increase exponentially with age in both gender, underscoring the impact of age on the hip fracture occurrences [10]. One meta-analysis showed that consuming more than 2 drinks a day has 1.39 times the risk of hip fracture than the abstainers [11]. The mono-infection of hepatitis C virus has been shown to be associated with higher risk for hip fracture than the controls with the relative risk highest among patients aged between 18∼39 years. The authors proposed that the elevated serum inflammatory cytokines in chronic hepatitis C virus carriers may activate the RANKL pathway-associated osteoclastogenesis, contributing to hip fracture [12]. According to a population-based twin study, stroke patients are associated with 5.09 times the risk of hip fracture than the subjects without stroke [13]. The decrease in muscle strength and postural stability after stroke may increase the risk for falls. Besides, immobilization increases the rate of bone loss and disuse osteoporosis. Both factors can increase the risk of fracture [14]. The consistent results demonstrated by our model not only supplement the published observations but also consolidate the validity and internal consistency of our model.

In our study, we demonstrated that the recipient has 2.71 times the risk for hip fracture than the matched controls. Compared with the previous series, our study provided the largest sample size and the longest follow-up duration for the recipients. We also identified that the recipients aged more than 65 years old at transplantation are 14.64 times (adjusted HR = 14.64, 95% CI = 1.47∼145) more likely to suffer from hip fracture than the recipients undergoing transplantation at the age of <45 years. We also showed that all of the recipients suffering from posttransplant hip fracture had the history of steroid use >30 days before the transplantation. The regimen of immunosuppressive agents did not significantly influence the occurrence of hip fracture among recipients. These findings have not been mentioned before and merit noticing.

There are limitations to our study. First, the extent of heterogeneity between the transplantation cohort and control cohort was a concern. However, excessive matching for the controls to reach homogeneity between the two cohorts will curtail the generalizability of the results. Second, the diagnosis of osteoporosis was coded by the physician if patient had the *T* score value <−2.5 in the bone densitometry assay. However, the bone densitometry survey is not ubiquitous in Taiwan; thus, we may miss some subjects with “silent osteoporosis.” Thirdly, although we excluded the subjects under antiresorptive and/or anabolic treatment in both groups, the status of vitamin D deficiency cannot be obtained.

## 5. Conclusions

Liver transplantation is associated with higher risk for hip fracture than the age-and-sex matched subjects. Age >65 years as well as pretransplant oral or systemic steroid use are associated with higher risk for posttransplant hip fracture. Preventive treatments, including antiosteoporotic medications, may be warranted for liver recipients who undergo transplantation at age >65 years and/or pretransplant steroid use.

## Figures and Tables

**Table 1:** Demographic profiles for the control and recipient cohorts.

Variable	Control cohort	Recipient cohort	*p* value
	*N* = 22010	*N* = 2201	
Age (year)	51.6 ± 9.8	51.7 ± 9.7	0.9371
Sex			>0.99
Female	5620 (25.5%)	562 (25.5%)	
Male	16390 (74.5%)	1639 (74.5%)	
Comorbidities			
CAD	3189 (14.5%)	292 (13.3%)	0.1192
DM	2508 (11.4%)	546 (24.8%)	<0.0001
Epilepsy	197 (0.9%)	29 (1.3%)	0.0494
Hypertension	6613 (30.0%)	721 (32.8%)	0.0083
Osteoporosis	1107 (5.0%)	140 (6.4%)	0.0071
Stroke	543 (2.5%)	70 (3.2%)	0.0422
ESRD	103 (0.5%)	28 (1.3%)	<0.0001
HBV infection	990 (4.5%)	1477 (67.1%)	<0.0001
HCV infection	350 (1.6%)	630 (28.6%)	<0.0001
Steroid	11858 (53.9%)	2061 (93.6)	

CAD: coronary artery disease; DM: diabetes mellitus; ESRD: end-stage renal disease; HBV: hepatitis B virus; HCV: hepatitis C virus infection.

**Table 2:** The risk factors for hip fracture analyzed by the Cox regression model.

Variables	Event	PYs	Rate	Crude HR 95% CI	Adjusted HR 95% CI
Transplantation					
No	70	93116	7.52	Ref	Ref
Yes	17	7910	21.49	2.88 (1.70–4.90)	2.71 (1.21–6.05)
Age group (years)					
<45	11	25593	4.30	Ref	Ref
45–64	59	69965	8.43	2.11 (1.11–4.03)	1.70 (0.87–3.30)
≧65	17	5467	31.1	8.40 (3.90–18.1)	5.21 (2.26–12.0)
Sex					
Female	25	25650	9.75	Ref	Ref
Male	62	75375	8.23	0.87 (0.55–1.39)	1.05 (0.64–1.72)
CAD					
No	67	87674	7.64	Ref	Ref
Yes	20	13351	15.0	2.06 (1.25–3.39)	1.10 (0.63–1.92)
DM					
No	65	89731	7.24	Ref	Ref
Yes	22	11294	19.5	2.79 (1.72–4.53)	1.66 (0.98–2.79)
Epilepsy					
No	85	100180	8.48	Ref	Ref
Yes	2	845	23.7	2.84 (0.70–11.5)	1.44 (0.33–6.18)
Hypertension					
No	47	73007	6.44	Ref	Ref
Yes	40	28019	14.3	2.33 (1.52–3.56)	1.33 (0.81–2.20)
Osteoporosis					
No	81	96345	8.41	Ref	Ref
Yes	6	4680	12.8	1.58 (0.69–3.61)	0.91 (0.37–2.20)
Stroke					
No	77	98853	7.79	Ref	Ref
Yes	10	2173	46.0	6.24 (3.22–12.1)	3.34 (1.62–6.87)
ESRD					
No	86	100555	8.55	Ref	Ref
Yes	1	471	21.25	2.53 (0.35–18.2)	0.99 (0.13–7.29)
HBV infection					
No	78	92224	8.46	Ref	Ref
Yes	9	8801	10.23	1.26 (0.63–2.52)	0.55 (0.24–1.26)
HCV infection					
No	75	97930	7.66	Ref	Ref
Yes	12	3095	38.77	5.38 (2.92–9.93)	2.64 (1.21–5.76)
Steroid					
No	36	46846	7.68	Ref	Ref
Yes	51	54180	9.41	2.88 (1.70, 4.90)	0.91 (0.57, 1.45)

PYs: person-years; Rate: incidence rate, per 10000 person-years; ref: reference for baseline. CI: confidence interval; CAD: coronary artery disease; DM: diabetes mellitus; ESRD: end-stage renal disease; HBV: hepatitis B virus; HCV: hepatitis C virus infection.

**Table 3:** Comparisons of liver recipients with and without hip fractures.

Variable	Liver transplant		Crude OR (95% CI)	Adjusted OR (95% CI)
	Hip fracture (−)	Hip fracture (+)		
	*N* = 2184 (%)	*N* = 17 (%)		
Age				
<45	475 (21.7)	1 (5.9)	Ref	Ref
45–64	1572 (72)	11 (64.7)	3.32 (0.43–25.8)	2.79 (0.34–22.65)
≧65	137 (6.3)	5 (29.4)	17.34 (2.01–149)	14.64 (1.47–145)
Sex				
Female	558 (25.5)	4 (23.5)	Ref	Ref
Male	1626 (74.5)	13 (76.5)	1.12 (0.36–3.43)	1.73 (0.49–6.13)
Comorbidities				
CAD	289 (13.2)	3 (17.6)	1.41 (0.40–4.92)	0.38 (0.04–3.19)
DM	539 (24.7)	7 (41.2)	2.14 (0.81–5.64)	1.38 (0.49–3.91)
Epilepsy	29 (1.3)	0 (0)	—	—
Hypertension	711 (32.6)	10 (58.8)	2.96 (1.12–7.81)	2.15 (0.73–6.30)
Osteoporosis	140 (6.4)	0 (0)	—	—
Stroke	69 (3.2)	1 (5.9)	1.92 (0.25–14.7)	1.80 (0.21–15.29)
ESRD	28 (1.3)	0 (0)	—	—
HBV infection	1469 (67.3)	8 (47.1)	0.43 (0.17–1.13)	0.43 (0.14–1.31)
HCV infection	621 (28.4)	9 (52.9)	2.83 (1.09–7.37)	1.99 (0.66–5.96)
Steroid	2044 (93.6)	17 (100.0)	—	—
Immunosuppressive agents				
Tacrolimus	167 (7.65)	1 (5.88)	0.76 (0.10–5.73)	0.46 (0.05–4.24)
Everolimus	31 (1.42)	1 (5.88)	4.34 (0.56–33.8)	2.63 (0.29–23.8)
MMF	366 (16.8)	3 (17.7)	1.06 (0.30–3.72)	1.32 (0.33–5.27)
Cyclosporin	81(3.71)	0 (0.00)	—	—

CI: confidence interval; CAD: coronary artery disease; DM: diabetes mellitus; ESRD: end-stage renal disease; HBV: hepatitis B virus; HCV: hepatitis C virus infection; MMF: mycophenolate mofetil.

## Data Availability

The data used to support the findings of this study are available from the corresponding author upon request.

## Abbreviations

CAD: Coronary artery disease
CI: Confidence interval
DM: Diabetes mellitus
ESRD: End-stage renal disease
HBV: Hepatitis B virus
HCV: Hepatitis C virus
HR: Hazard ratio
LT: Liver transplantation
MMF: Mycophenolate mofetil
NHI: National health insurance.

## References

[1] Luca L., Westbrook R., Tsochatzis E. A. (2015). Metabolic and cardiovascular complications in the liver transplant recipient. *Annals of Gastroenterology: Quarterly Publication of the Hellenic Society of Gastroenterology*.

[2] Compston J. (2003). Osteoporosis after liver transplantation. *Liver Transplantation*.

[3] Ramsey-Goldman R., Dunn J. E., Dunlop D. D. (1999). Increased risk of fracture in patients receiving solid organ transplants. *Journal of Bone and Mineral Research*.

[4] Monegal A., Navasa M., Guañabens N. (2001). Bone disease after liver transplantation: a long-term prospective study of bone mass changes, hormonal status and histomorphometric characteristics. *Osteoporosis International*.

[5] Premaor M. O., Das T. K., Debiram I. (2011). Fracture incidence after liver transplantation: results of a 10-year audit. *QJM*.

[6] Sattui S. E., Saag K. G. (2014). Fracture mortality: associations with epidemiology and osteoporosis treatment. *Nature Reviews Endocrinology*.

[7] Leidig-Bruckner G., Hosch S., Dodidou P. (2001). Frequency and predictors of osteoporotic fractures after cardiac or liver transplantation: a follow-up study. *The Lancet*.

[8] Guichelaar M. M. J., Schmoll J., Malinchoc M., Hay J. E. (2007). Fractures and avascular necrosis before and after orthotopic liver transplantation: long-term follow-up and predictive factors. *Hepatology*.

[9] Hsu C. C., Hsu H. C., Lin C. C. (2018). Increased risk for hip fractures among patients with cholangitis: a nationwide population-based study. *BioMed Research International*.

[10] Cooper C., Melton L. J. (1992). Epidemiology of osteoporosis. *Trends in Endocrinology & Metabolism*.

[11] Berg K. M., Kunins H. V., Jackson J. L. (2008). Association between alcohol consumption and both osteoporotic fracture and bone density. *The American Journal of Medicine*.

[12] Lo Re V., Volk J., Newcomb C. W. (2012). Risk of hip fracture associated with hepatitis C virus infection and hepatitis C/human immunodeficiency virus coinfection. *Hepatology*.

[13] Sennerby U., Melhus H., Gedeborg R. (2009). Cardiovascular diseases and risk of hip fracture. *JAMA*.

[14] Kanis J., Oden A., Johnell O. (2001). Acute and long-term increase in fracture risk after hospitalization for stroke. *Stroke*.

